# Development and psychometric properties of the Japanese version of the capacity for wonder scale

**DOI:** 10.5116/ijme.6819.fcae

**Published:** 2025-05-29

**Authors:** Hirohisa Fujikawa, Takayuki Ando, Kayo Kondo, Mikio Hayashi

**Affiliations:** 1 Center for General Medicine Education, School of Medicine, Keio University, Shinjuku-ku, Tokyo, Japan; 2 School of Modern Languages and Cultures, Durham University, Durham, UK; 3 Center for Health Professions Education, Kansai Medical University, Hirakata, Osaka, Japan

**Keywords:** Capacity for wonder, medical student, factor analysis

## Abstract

**Objectives:**

To develop a Japanese version of the capacity for wonder Scale (J-CfWS) and to examine its psychometric properties.

**Methods:**

An anonymous online self-administered questionnaire was distributed to medical undergraduates in three universities in Japan. We assessed the structural (factor analysis and model fitness test (comparative fit index, root mean square error of approximation, and standardized root mean square residual)) and convergent validity and internal consistency reliability of the scale.

**Results:**

384 participants were included in the analysis. We employed a split-half validation approach, with exploratory factor analysis (EFA) on one half and confirmatory factor analysis (CFA) on the other. EFA led to a 9-item scale with a three-factor structure. CFA supported this three-factor structure with good model fitness indices (comparative fit index = 0.986, root mean square error of approximation = 0.036, and standardized root mean square residual = 0.036). The Pearson correlation coefficient between J-CfWS total scores and the Epistemic Curiosity Scale total scores was significant, r(382) = .60, p< .001, indicating a positive correlation between the two variables. The internal consistency reliability was good, with an overall Cronbach’ alpha of 0.82.

**Conclusions:**

The J-CfWS was developed. We confirmed its psychometric properties. It will be useful in assessing the impact of curricula aimed at cultivating CfW among medical trainees (e.g. arts and humanities courses). It can also be useful to researchers who wish to verify the association between CfW and other concepts.

## Introduction

Philosophers and scientists alike insist that wonder is the central virtue and engine of all education.^1^^-^^3^ Wonder is defined as “a feeling of radical appreciation for a triggering event, typically accompanied by a behavior of pausing to reflect and a motivation to reorient one’s self-understanding and sense of the world.”^4^ Although curiosity seems to be related to wonder, they differ in the following ways. Curiosity is a goal-oriented interest in answering a specific question and is superficial, whereas wonder is broader, more deeply emotional, and comes from within.^4^^,^^5^ Accordingly, wonder is at the origin of learning,^2^ and experience of wonder is irreplaceable and should be protected and promoted in academic settings.

Thus, the concept of wonder has been the subject of philosophical and social scientific inquiry and has been overlooked in the health professions education.^6^ However, in 2012, Evans indicated the importance of fostering capacity for wonder (CfW) among healthcare professionals and trainees.^7^ In 2018, Geller and colleagues posited that the adverse pressures inherent in the learning environment of medical education (i.e., the pervasive competitive atmosphere) can impede the growth of essential personal virtues for healthcare professionals, which encompass respect, compassion, altruism, recognizing ambiguity/uncertainty, and acknowledging mistakes.^8^ They asserted that fostering CfW is crucial to counteract those detrimental influences.^8^ Since 2020, research has been conducted on the CfW in the field of medical education, particularly in the U.S.^9^^,^^10^ Wonder is a personal resource, a source of renewal for the physician that fuels the diagnostic imagination, and a timely and valuable reminder of the embodied agency of both patient and physician, with a diminution of one’s self and an orientation towards humility.^7^^,^^11^ The CfW can therefore promote ethical leadership and lifelong learning and help maintain important qualities such as tolerance of ambiguity and the fostering of empathy and humility.^7^^-^^9^^,^^12^ In light of these, development of the capacity for wonder among physicians and medical trainees is of high priority.

Effective education in cultivating CfW first necessitates a tool for measuring it. A measure for assessing CfW was not available until the CfW Scale (CfWS) was developed by Geller and colleagues in 2020.^4^  The scale was validated among undergraduate students at a research university in the U.S., and then examined among medical students.^9^ Currently, in the U.S., the CfWS is widely used to assess the CfW of medical students. For example, Tackett and colleagues implemented an arts-based elective for medical students and evaluated its educational effectiveness by using the CfWS.^10^ Zheng and colleagues described the usefulness of the scale in the quantitative evaluation of arts and humanities activities.^6^

In contrast, in medical education in Japan, few educational interventions have been implemented to foster the CfW and develop its assessment. The primary reason is that no scale has been developed in Japan to measure the CfW. Given the potential of wonder to promote humanistic care and lifelong learning, its development is essential, and thus, the development of a scale for measuring it is urgent. However, it is unclear whether the CfWS can be directly applied in the Japanese medical education context because the CfW indeed vary across cultures and can be influenced by educational systems and societal norms. Cultural background would play an important role in shaping CfW, and tools for measuring CfW should be developed with respect to cultural context.

Accordingly, the aim of this study was to translate and culturally adapt the CfWS for use in Japan and to examine its psychometric properties.

## Methods

### Design, setting, and participants

This study was conducted under a multicentered cross-sectional design in May 2024 as part of a series of studies exploring the professionalism of medical students. Given that the CfWS comprises 10 items, that factor analysis requires a sample size 10 times the number of items,^13^ and that we employed the split-half validation approach (as will hereinafter be described in detail), we determined that a final sample size of over 200 would be optimal. Considering that the response rate for previous online questionnaire studies targeting medical students in Japan was approximately 10%,^14^^,^^15^ and that there are around 700 medical students per medical school in Japan, we decided to recruit medical students from three medical schools. We invited medical undergraduates at three universities in Japan, selected based on their differing location (Kanto, Kyushu, and Chubu regions) and type (private and public), to participate through the medical education director of each university. The request for participation in the study was disseminated to medical students by the medical education directors of each university via a mailing list or electronic bulletin board system. Prior to participation, we informed them of the voluntary nature and anonymization of the study. Only those who agreed to participate were included.

Participants were asked to answer an online anonymous self-administered questionnaire on SurveyMonkey (www.surveymonkey.com). Non-respondents were reminded to complete the survey several times via email. A 3,000-yen gift card was provided to 10 drawing winners. This study was performed according to the ethical standards and principles of the Declaration of Helsinki. Ethical approval was obtained from the ethics committee of Keio University School of Medicine (20231223).

### Measures

#### Original CfW scale

In the early 2020’s, Geller and colleagues developed the 10-item CfWS and examined its psychometric properties.^4^^,^^9^ It has the following 2 dimensions: perspective shifting (Q1–5) and emotional reawakening (Q6–10).^4^^,^^9^ The 10 items are rated on 6-point Likert scales ranging from 1 (not at all likely) to 6 (extremely likely).^4^^,^^9^ The score of the CfWS was created by simply summing up the responses to each item, with higher scores indicating greater CfW.

### Procedure for translation

Here, we described the translation process of the scale, conducted in accordance with the seven-step cross-cultural adaptation process of Beaton and colleagues.^16^ The necessity for this process arises from the fact that items need to be translated accurately and adapted culturally. This is important to demonstrate the scales to be used across cultures and to maintain the content validity of the measure at the conceptual level.^16^^-^^20^

First, we emailed the original author (GG) of the English version of the scale, who readily agreed to our development of a Japanese version.

Second, forward translation was performed. Three translators (HF, TA, and KK) independently translated the scale from English into Japanese. All three translators are native Japanese speakers who are fluent in English and are familiar with both Japanese and American cultures. HF and KK have extensive experience in developing Japanese translations of scales in the field of health professions education.^15^^,^^21^^,^^22^

Third was synthesis of the translated versions by HF, TA, and KK. The proposed translations were compared with each other and thoroughly discussed until a consensus was reached (Ver. 1).

Fourth, the three translators asked a professional bilingual translator who was not involved in the study to translate Ver. 1 back from Japanese into English. HF, TA, and KK compared the back-translated version with GG’s original version. Repeated discussions led to further refinement of Ver. 1; all authors proofread it and prepared a new version (Ver. 2).

Fifth, the three translators asked an expert in health professions education (MH) to review Ver. 2. The expert provided advice on the equivalence of meanings and expressions. In accordance with his advice, it was revised (Ver. 3).

Sixth, we asked GG to review Ver. 3 for any deviation from the original English version. Based on GG’s feedback, further proofreading was done (Ver. 4).

Seventh, a pilot test was conducted with three medical trainees to check for problems with the clarity of expression and meaning. The test showed no major problematic items following the translation process, and Ver. 4 was therefore considered the final version. The scale’s face and content validity were confirmed by all authors.

### Statistical analysis

We examined the structural validity of the Japanese version of the CfWS (J-CfWS) through exploratory factor analysis (EFA) and confirmatory factor analysis (CFA). We decided to perform EFA followed by CFA because, although there is a possibility that the cultural differences between Japan and the U.S. may affect the factor structure, the aim of this study was to develop a scale optimized for the Japanese medical education context. Given the issues with conducting both EFA and CFA on the same sample,^23^ we randomly divided the sample into two groups, one for EFA and the other for CFA.

Prior to EFA, Kaiser–Meyer–Olkin (KMO) measurement and Bartlett’s test of sphericity were used to assess sampling adequacy for factor analysis. Factor analysis requires a KMO value greater than 0.60 and a significant Bartlett’s test.^24^ The maximum likelihood EFA with promax rotation was then employed on the half-sample. We decided to choose promax rotation because it allows for correlated factors, and we expected the constructs of the scale to be correlated. We performed parallel analysis to determine the number of factors.^24^ Only items with factor loadings above 0.35 were retained.

Next, we performed CFA on the other half-sample to assess the suitability of the factor structure (three-factor model, as will hereinafter be described in detail) suggested by the EFA and compared the model fitness of the three-factor model with several other models (a two-factor model similar to the original English version and a single-factor model). We assessed model fitness by calculating comparative fit index (CFI), root mean square error of approximation (RMSEA), and standardized root mean square residual (SRMR). CFI is utilized to analyze the model’s fit goodness. RMSEA is used for the parsimonious fit index. SRMR shows the error amount resulting from evaluation of the specified model and is used as the absolute fit index.^25^ Acceptable criteria are a CFI > 0.90, an RMSEA < 0.08, and an SRMR < 0.08.^24^^-^^26^

Convergent validity was examined through hypothesis testing. Since CfW appears to be associated with curiosity,^4^^,^^5^ we used the Pearson correlation coefficient between the J-CfWS total scores and the Epistemic Curiosity Scale total scores to examine validity. The Epistemic Curiosity Scale has 12 items; respondents rate each item on a 5-point Likert scale ranging from “not at all” (score of 1) to “extremely” (score of 5).^27^ Scores are calculated by summing the responses of the items, with higher scores indicating higher curiosity. A Pearson correlation coefficient value greater than 0.30 is deemed acceptable.^28^

We assessed the internal consistency reliability of the J-CfWS by using Cronbach’s alpha on the whole sample. Previous literature suggested the following criteria: < 0.50, insufficient; 0.50–0.69, moderate; 0.70–0.79, satisfactory; and ³ 0.80, good.^29^ Finally, descriptive statistics were conducted for the J-CfWS scores. We chose complete case analysis. Our statistical analysis was performed using R version 4.4.0.

## Results

The number of eligible participants was 2170, of whom 399 answered the questionnaire. After exclusion of 15 participants with missing data, 384 (17.7%) were included in the analysis. Table 1 summarizes the characteristics of the participants. The majority were male 238 (62.0%). Regarding academic year, the most common group were 4th-year students 127 (33.1%), followed by 1st-year students 90 (23.4%).  Table 2 shows the participants’ responses to each item of the questionnaire.

**Table 1:** Participants’ characteristics (N = 384)

Characteristics		n (%)
Gender		
	Female	144 (37.5)
	Male	238 (62.0)
	Others	2 (0.5)
Year		
	1st	90 (23.4)
	2nd	32 (8.3)
	3rd	48 (12.5)
	4th	127 (33.1)
	5th	52 (13.5)
	6th	35 (9.1)

### Structural validity

First, EFA was conducted on 190 participants. The KMO value was 0.83, indicating that it was appropriate to perform factor analysis on this dataset. Also, the Bartlett’s test was significant, c^2^ (45) = 580.759, p < .001. We then performed EFA, with exclusion of 1 of the 10 items, “4. Find yourself pausing to reflect.” The item was excluded on the grounds that it exhibited a low factor loading (< 0.30) and appeared to be incompatible with the Japanese context of CfW.

**Table 2:** Responses to the questionnaire (N = 384)

Original English item	Responsesn (%)					
	1 = Not at all likely	2	3	4	5	6 = Extremely likely
1. Find yourself drawing new connections between things in the world	23 (6.0)	56 (14.6)	74 (19.3)	98 (25.5)	84 (21.9)	49 (12.8)
2. Take to heart experiences that challenge your understanding of the world	18 (4.7)	47 (12.2)	67 (17.4)	106 (27.6)	96 (25.0)	50 (13.0)
3. Be described by others as inquisitive	33 (8.6)	72 (18.8)	68 (17.7)	99 (25.8)	73 (19.0)	39 (10.2)
4. Find yourself pausing to reflect	11 (2.9)	56 (14.6)	84 (21.9)	113 (29.4)	74 (19.3)	46 (12.0)
5. Move among several different perspectives on the same situation like a camera or microscope lens zooming in and out	21 (5.5)	63 (16.4)	77 (20.1)	114 (29.7)	72 (18.8)	37 (9.6)
6. Experience familiar things as if for the first time	41 (10.7)	123 (32.0)	84 (21.9)	83 (21.6)	35 (9.1)	18 (4.7)
7. Feel amazement during the ordinary course of events	19 (4.9)	63 (16.4)	67 (17.4)	122 (31.8)	76 (19.8)	37 (9.6)
8. Feel personally engaged by an experience that takes your breath away	15 (3.9)	32 (8.3)	50 (13.0)	108 (28.1)	100 (26.0)	79 (20.6)
9. See the world with an interest of a child	12 (3.1)	43 (11.2)	80 (20.8)	109 (28.4)	79 (20.6)	61 (15.9)
10. Experience surprise	8 (2.1)	29 (7.6)	47 (12.2)	126 (32.8)	107 (27.9)	67 (17.4)

The research team reviewed this item and concluded that it should be deleted. The final solution was a three-factor structure. After iterative discussions among the research team, these factors were named as follows: Factor 1 (perspective shifting, 4 items), Factor 2 (experiencing the uncharted, 3 items), and Factor 3 (emotional reawakening, 2 items) (Table 3).

**Figure 1:**
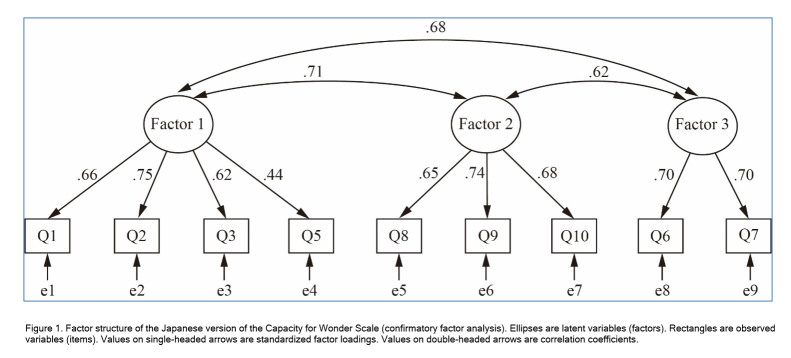
Factor structure of the Japanese version of the Capacity for Wonder Scale (confirmatory factor analysis). Ellipses are latent variables (factors). Rectangles are observed variables (items). Values on single-headed arrows are standardized factor loadings. Values on double-headed arrows are correlation coefficients.

Second, we performed CFA on the remaining 194 participants. Table 4 shows the goodness-of-fit results. As the table indicates, compared to all other models, the three-factor model suggested by the EFA provided the best model fit. All fit indices met their respective criteria, namely CFI = 0.986, RMSEA = 0.036, and SRMR = 0.036. Figure 1 shows the path diagram.

### Convergent validity

The Pearson correlation coefficient between the J-CfWS total scores and Epistemic Curiosity Scale total scores was computed. There was a positive correlation between the two variables, r(382) = .60, p < .001.

### Internal consistency reliability and descriptive statistics

The internal consistency reliability of the 9-item scale was good, with Cronbach’s alpha = 0.82. Descriptive statistics are shown in Table 5. Thus, we obtained the final version of the J-CfWS.

## Discussion

In this study, we developed the J-CfWS and examined its psychometric properties. Our translation into Japanese was performed by experienced translators in the field of health professions education, in accordance with an international guideline.^16^ To our knowledge, the J-CfWS is the first scale for measuring CfW in Japan.

In this study, as with the English version,^4^ our analysis supported the good structural validity, convergent validity, and internal consistency reliability of our Japanese version. In addition, the strength of the study is the robustness of its translation process. We translated the original scale with reference to a cross-cultural instrument adaptation guideline in the following steps: forward translation, synthesis, back-translation, expert review, and pilot testing.^16^ This rigorous translation process strengthened the validity of the scale.

Factor analysis revealed that the J-CfWS had a different factor-structure to the original English version: the former had a three-factor structure, while the latter had two factors. Similarly, previous cross-cultural validation studies in the field of Japanese medical education have also identified a factor structure that differs from the original English version, as a result of factor analysis. In their study of the development of the Japanese version of the interprofessional facilitation scale, Haruta and colleagues did not identify the factor of “contextualizing interprofessional education,” which had been identified in the recent Canadian study.^30^^,^^31^ Rather, the authors did extract a factor of “respect for each professional.”^30^ They concluded that this divergence was likely caused by characteristics of the Japanese culture, as based on relationalism.^30^ Factor analysis extracted four factors in the development of the Japanese translation of the Patient Care Ownership Scale, while the original English version had three factors.^22^^,^^32^ The research team suggested that the discrepancy might be related to a unique cultural feature of Japan, namely, a historical code of personal conduct (“Bushido”).^22^^,^^33^ In our study, the precise mechanism behind the differences in factor structure between the Japanese and English versions remains unclear. Nevertheless, given that CfW is expected to be influenced by culture,^4^ participant responses to the questionnaire may have been affected by cultural differences between the U.S. and Japan. The U.S. culture frequently encourages individualism and assertiveness, potentially encouraging more open expression of wonder. In collectivist cultures, there may be a greater emphasis on maintaining harmony and conformity, which can influence the manner in which individuals express wonder.

**Table 3:** Results of the exploratory factor analysis of the Japanese version of the Capacity for Wonder Scale (N = 190)

Items (as in original English version)	Factor loading		
	Factor 1	Factor 2	Factor 3
1. Find yourself drawing new connections between things in the world	0.76	-0.01	0.01
2. Take to heart experiences that challenge your understanding of the world	0.60	0.12	-0.09
3. Be described by others as inquisitive	0.75	0.04	0.01
5. Move among several different perspectives on the same situation like a camera or microscope lens zooming in and out	0.52	-0.09	0.07
6. Experience familiar things as if for the first time	0.12	0.02	0.36
7. Feel amazement during the ordinary course of events	-0.09	-0.01	1.04
8. Feel personally engaged by an experience that takes your breath away	0.03	0.84	-0.11
9. See the world with an interest of a child	0.28	0.55	-0.02
10. Experience surprise	-0.16	0.71	0.24
	Value		
Eigenvalue	3.23	0.52	0.27
Percentage variance explained	21	18	14

**Table 4:** Results of the confirmatory factor analysis

Model	CFI	RMSEA	SRMR
One-factor model^a^	0.880	0.099	0.059
Two-factor model^b^	0.927	0.079	0.052
Three-factor model^c^	0.986	0.036	0.036
Acceptable criteria	> 0.90	< 0.08	< 0.08

Abbreviations: CFI, comparative fit index; RMSEA, root mean square error of approximation; SRMR, standardized root mean square residual

a Factor 1: Q1, Q2, Q3, Q5, Q6, Q7, Q8, Q9, Q10

b Factor 1: Q1, Q2, Q3, Q5; Factor 2: Q6, Q7, Q8, Q9, Q10 (similar to the original English version)

c Factor 1: Q1, Q2, Q3, Q5; Factor 2: Q8, Q9, Q10; Factor 3: Q6, Q7 (suggested by the exploratory factor analysis)

**Table 5:** Descriptive statistics of the Japanese version of the Capacity for Wonder Scale (N = 384)

	Number of items	Mean	SD	Observed range	Cronbach’s alpha
Total	9	34.32	7.82	9–54	0.82
Factor 1 (perspective shifting)	4	15.03	4.17	4–24	0.73
Factor 2 (experiencing the uncharted)	3	12.55	3.22	3–18	0.76
Factor 3 (emotional reawakening)	2	6.74	2.27	2–12	0.62

Japan takes a middle position on the dimension of individualism/collectivism.34 The discrepancy in individualism/collectivism between the U.S. and Japan may have influenced the responses of the participants to the questionnaire. Further studies are needed to clarify the mechanism of this difference. Our developed measure will contribute to various settings and enhance the quality of health professions education. For example, it can be useful as a scale for assessing the educational interventions of curricula that aim to nurture CfW among medical trainees. Zheng and colleagues indicated that learning activities in arts and humanities subjects could nurture physicians’ CfW.^6^ In particular, visual thinking strategies could be readily and effectively integrated into medical education for cultivating CfW.^6^ It will also be useful as a research tool to examine the association between CfW and other concepts (e.g., academic performance and empathy) in the field of medical education. Medical learners with greater CfW may foster a heightened sensitivity to nuance, an attitude that admires deep contemplation and radical appreciation, and deeper pursuit for meaning.^6^ This can ultimately contribute to enhanced patient care, scientific discovery, and lifelong learning.^7^ Thus, the CfWS has the potential to be used in a range of medical education settings and research contexts, with the possibility of enhancing the quality of medical education and patient care.

Potential limitations of this study should also be noted. First, the number of institutions in which the validation survey was performed was relatively small. Further studies which recruited larger numbers of institutions would strengthen the robustness of the J-CfWS. Second, the response rate was relatively small, which may raise questions about representativeness. The voluntary nature of the study, which was recruited via mailing lists or electronic bulletin board system, and the use of an online survey format,^35^ may have contributed to the lower response rate. Efforts were made to optimize the response rate, including the use of reminder emails and remuneration.

Figure 1. Factor structure of the Japanese version of the Capacity for Wonder Scale (confirmatory factor analysis). Ellipses are latent variables (factors). Rectangles are observed variables (items). Values on single-headed arrows are standardized factor loadings. Values on double-headed arrows are correlation coefficients.

Third, although our study used split-half validation approach, EFA and CFA were conducted on the same dataset, which might introduce bias. However, this is an acceptable method, commonly used in previous exploratory studies.^15^^,^^21^^,^^36^ Future research should use independent samples for EFA and CFA to validate the findings further. Fourth, we should acknowledge that the three-factor structure identified in this study may not be final. The three factors were named based on discussions among researchers, rather than from literature or theory. Finally, psychometric properties other than structural validity, convergent validity, and internal consistency reliability were not examined. Future studies should test the three-factor model across different samples to confirm its stability and examine other psychometric properties (e.g., test-retest reliability and discriminant validity), which would also strengthen the developed scale.

## Conclusions

We translated and adapted the CfWS into Japanese and examined its psychometric properties. The scale will be useful in evaluating the impact of the curricula aimed at cultivating CfW among medical trainees (e.g., arts and humanities courses). It can benefit medical education researchers who aim to uncover the relationship between CfW and other concepts. Thus, the results of this study and the measure developed will provide helpful information to faculty members and researchers aiming to improve the medical education system by enhancing CfW among medical students.

### Acknowledgments

The authors wish to thank all participants.

This work was partly supported by JSPS KAKENHI Grant Number 23K19809 and 24K20148.

### Conflict of Interest

The author declares that there is no conflict of interest.
